# From Traditional Usage to Pharmacological Evidence: Systematic Review of* Gunnera perpensa* L.

**DOI:** 10.1155/2016/1720123

**Published:** 2016-12-07

**Authors:** Alfred Maroyi

**Affiliations:** Department of Botany, University of Fort Hare, Private Bag X1314, Alice 5700, South Africa

## Abstract

*Gunnera perpensa* is the only species of the genus* Gunnera* that has been recorded in Africa. Its leaves, rhizomes, roots, and stems are reported to possess diverse medicinal properties and used to treat or manage various human and animal diseases and ailments.* Gunnera perpensa* is an ingredient in many herbal concoctions and prescriptions which have been used to induce or augment labour, postnatal medication, to treat parasitic diseases, urinary complaints, kidney problems, general body pains, sexually transmitted infections, and many other diseases. Several classes of phytochemicals including alkaloids, benzoquinones, ellagic acids, flavonoids, phenols, proanthocyanidins, tannins, and minerals have been isolated from* G. perpensa*. Scientific studies on* G. perpensa *indicate that it has a wide range of pharmacological activities including acetylcholinesterase, anthelmintic, antibacterial, antifungal, antinociceptive, anti-inflammatory, antioxidant, antitumour, lactogenic, and uterotonic.* Gunnera perpensa* has a lot of potential as a possible source of pharmaceutical products for the treatment of a wide range of both human and animal diseases and ailments. Some of the chemical compounds isolated from* G. perpensa *have demonstrated various biological activities when investigated in* in vitro* assays. Future research should focus on the mechanisms of action of the isolated compounds, their efficacy, toxicity, and clinical relevance.

## 1. Introduction

*Gunnera perpensa* L. is a member of the genus* Gunnera* L., a single genus of the family Gunneraceae. The genus was named in honour of the Norwegian bishop and botanist Johan Ernst Gunnerus (1718–1773) [1]. According to Qiu et al. [2] and Soltis et al. [3], there is a close relationship between genus* Gunnera* and* Myrothamnus flabellifolia* Welw., a morphologically different taxon confined to dry habitats belonging to the monogeneric family Myrothamnaceae. This sister relationship between genera* Gunnera* and* Myrothamnus *was inferred by rbcL, atpB, and 18S molecular data with a bootstrap value of 75% [3] and also by rbcL and rps16 introns molecular data [4]. Research by Moore et al. [5] revealed that* G. perpensa* and* M. flabellifolia* have similar geographical distribution. Genus* Gunnera* includes 30–40 herbaceous species, mostly distributed in the southern hemisphere [4].* Gunnera manicata* Linden ex Delchev. and* G. tinctoria* (Molina) Mirb. are among some of the popular* Gunnera* species in the world [6–8]. They are natives of South America but also widely cultivated in temperate, tropical, and subtropical countries as ornamental and medicinal plant species [1, 6–11]. Both species have escaped from cultivation in some countries; for example, these species are now naturalized and considered potential invasive aliens in Australia, Ireland, and New Zealand [8, 12]. Aqueous and methanolic extracts of* G. manicata* displayed antioxidant and antimicrobial activities [9] corroborating wide usage of the species as herbal medicines in Brazil.* Gunnera tinctoria* is widely used as herbal medicine for analgesic, anti-inflammatory, cardiovascular, gastrointestinal, genitor-urinary, obstetric-gynaecological, and respiratory diseases in Argentina [13]. In Sub-Saharan Africa,* G. perpensa *is the most popular* Gunnera* species.* Gunnera perpensa* was the first species of the genus to be described by Linnaeus in 1767 and exists in Africa from Sudan, Ethiopia, Democratic Republic of Congo (DRC), Burundi, Madagascar, Rwanda, Uganda, Kenya, Tanzania, Botswana, Namibia, Zimbabwe, Mozambique, Lesotho, South Africa, and Swaziland [14].* Gunnera perpensa* is widely known for its high medicinal importance in several traditional medicine systems in southern Africa which resulted in the creation of some formulas or prescriptions (Table 1). Many of these formulas or prescriptions are in clinical use, usually prepared as decoctions or infusions, and sold in informal markets, medicinal herbal (muthi) markets, supermarkets, and pharmacies.  Table 2 shows how* G. perpensa* is used alone in monotherapeutic applications.* Gunnera perpensa *has long been used in traditional medicine by different ethnic groups in southern Africa as a remedy to initiate labour, ensure easy childbirth, and facilitate the expulsion of placenta and clearing of the womb after birth in both women and animals [15–27].* Gunnera perpensa* is an important ingredient of at least three traditional concoctions in South Africa, known as “*imbiza ephuzwato,*” “*inembe,*” and “*isihlambezo*” (Table 1).* Imbiza ephuzwato* is a herbal tonic made from a mixture of roots, bulbs, rhizomes, and leaves of 21 different medicinal plant species used as a multipurpose remedy (Table 1).* Inembe* is a potent labour-inducing herbal mixture taken regularly during pregnancy to ensure easy childbirth, but it is often used as an abortifacient. It is made up of roots of* Cyphostemma natalitium* (Szyszyl.) J. J. M. van der Merwe,* Rhoicissus tridentata* subsp.* cunefolia* (Eckl. & Zeyh.) Urton, and* Triumfetta rhomboidea* Jacq. mixed with rhizomes* G. perpensa* [23].* Isihlambezo* is a herbal decoction used by many pregnant women in South Africa as a pregnancy tonic to hasten childbirth and expel placenta.* Isihlambezo* decoctions are also administered to animals by the Zulu and southern Sotho people in South Africa to assist in the expulsion of the placenta [15, 16, 27].

The leaves, rhizomes, roots, and stems of* G. perpensa *are reported to possess diverse medicinal properties and used to treat or manage various human and animal diseases and ailments throughout the distributional range of the species (Tables 1 and 2). In most instances, the roots or rhizomes of* Gunnera perpensa *are preferred and taken orally as decoction, infusion, or tincture (Tables 1 and 2). Decoctions or infusions of the root or rhizome are used for abdominal pain, bladder problems, body cleansing, cancer, colds, earache, endometritis, gastrointestinal parasites, gonorrhoea, heart diseases, hypertension, impotence, infertility, kidney problems, poor appetite, rheumatic pains, scabies, syphilis, and urinary infections [24, 27–34]. Root decoction of* G. perpensa *is used by Zulu traditional healers in South Africa to stimulate milk production [35]. A decoction of the rhizomes of* G. perpensa *is applied as a dressing for wounds and psoriasis [16, 36]. The resource-limited farmers in the Eastern Cape province, South Africa, use* G. perpensa *as an alternative control of gastrointestinal parasites in village chickens [31, 37, 38]. The Xhosa people in the Eastern Cape province, South Africa, boil* G. perpensa *stem with water and take a glass of decoction as remedy for constipation [39]. In South Africa, the root decoction is taken for colds and as a tonic for abdominal pain, indigestion, poor appetite, a bleeding stomach, rheumatic fever, and scabies. In the KwaZulu Natal province, South Africa,* G. perpensa *leaves are collected from the wild and used as a leafy vegetable, locally known as “*imifino” *in Zulu [40]. The Venda people in the Limpopo province, South Africa, collect fresh leaves and cook them as leafy vegetables mixed with other indigenous or traditional leafy vegetable species [41]. Similarly, in Swaziland, the roots, stalks, and stems are edible and also used as ingredients of traditional beer [42]. In Lesotho,* G. perpensa *leaves are used as hot poultices for wounds and boils [28, 43], decoction of roots is used to regulate menstrual periods and as remedy for menstrual pains [43, 44] and as colic in pregnant women [43, 44], expulsion of placenta in both women and animals [18], and vermifuge in humans and animals [28], and leaves are burnt, crushed, and smoked for headaches [43].

*Gunnera perpensa *is a robust, erect, perennial herb which grows up to 1 m tall and always grows in moist habitats, marshy areas, and along river banks. Its roots are 30 cm thick, fleshy, dark brown or blackish on the outside but yellow or pinkish-red inside [1, 45]. All the leaves arise from a central tuft near the top of the apex, just above the soil level. The leaves are large, dark bluish-green, and kidney shaped and covered with hairs on both surfaces especially along the veins, in young leaves. The margins of the leaves are irregularly toothed. The veins are very noticeable on the lower surface of the leaf, radiating from the point where the petiole joins the leaf, referred to as palmate radiation [1, 45]. The flowers are numerous, small and not very noticeable, pinkish, reddish brown, and borne on a long slender spike, which is taller than the leaves. There will be female flowers at the base, male flowers at the top, and bisexual flowers in the middle of each spike [1, 45]. It is unable to tolerate frost and cold conditions [45]. In southern Africa,* G. perpensa *is often referred to as “river pumpkin” in English, “gobho” in Swati, “iphuzilomlambo” in Xhosa, and “ugobho” in Zulu.

Like most medicinal plants in southern Africa,* G. perpensa* is collected from the wild. The unsustainable harvesting of* G. perpensa *as herbal medicine and destruction of its wetland habitat due to development and agriculture are threatening its continued existence. Although* G. perpensa *is widespread throughout its distributional range, its population is declining due to overexploitation of its rhizomes and roots which are sold in the medicinal (*muthi*) markets throughout South Africa. According to Dold and Cocks [46],* G. perpensa* is heavily traded in the Eastern Cape province, South Africa, characterized by a high price on the medicinal (*muthi*) market with 1 kg fetching R47.80 (US$4.54) with 115.6 kilogrammes as mean quantity traded per trader per annum. In Swaziland,* G. perpensa *is a dominant medicinal plant harvested from wetlands, with traders generating R150–200 (US$20.49–27.32) monthly from selling the rhizomes or roots of the species [28]. Furthermore, research by Williams [47] revealed that large volumes of this species are traded in medicinal (*muthi*) markets in South Africa and local extirpations have been noted particularly in the Eastern Cape [46] and KwaZulu Natal provinces [26, 48]. Raimondo et al. [49] categorized* G. perpensa *as declining in South Africa using the modified IUCN Red List Categories and Criteria version 3.1 of threatened species [50–52]. According to Victor and Keith [51] and von Staden et al. [52], a species categorized as Least Concern (LC) under the IUCN Red List Categories and Criteria version 3.1 [50] can additionally be categorized either as rare, critically rare, or declining. The observed population decline of* G. perpensa *in Lesotho and South Africa [49, 53] is due to overexploitation as herbal medicine, destruction of its habitat, medicinal plant trade, and popularity of the species in the medicinal (*muthi*) markets. It is within this context that the current study was carried out, aimed at discussing how* G. perpensa* is used as a single agent or in complex herbal mixtures, and assesses the phytochemistry and pharmacology of the species. The review is also aimed at assessing whether there is correlation between the ethnomedicinal uses of* G. perpensa *with its chemical and bioactive properties.

## 2. Phytochemistry

**Figure 1:**
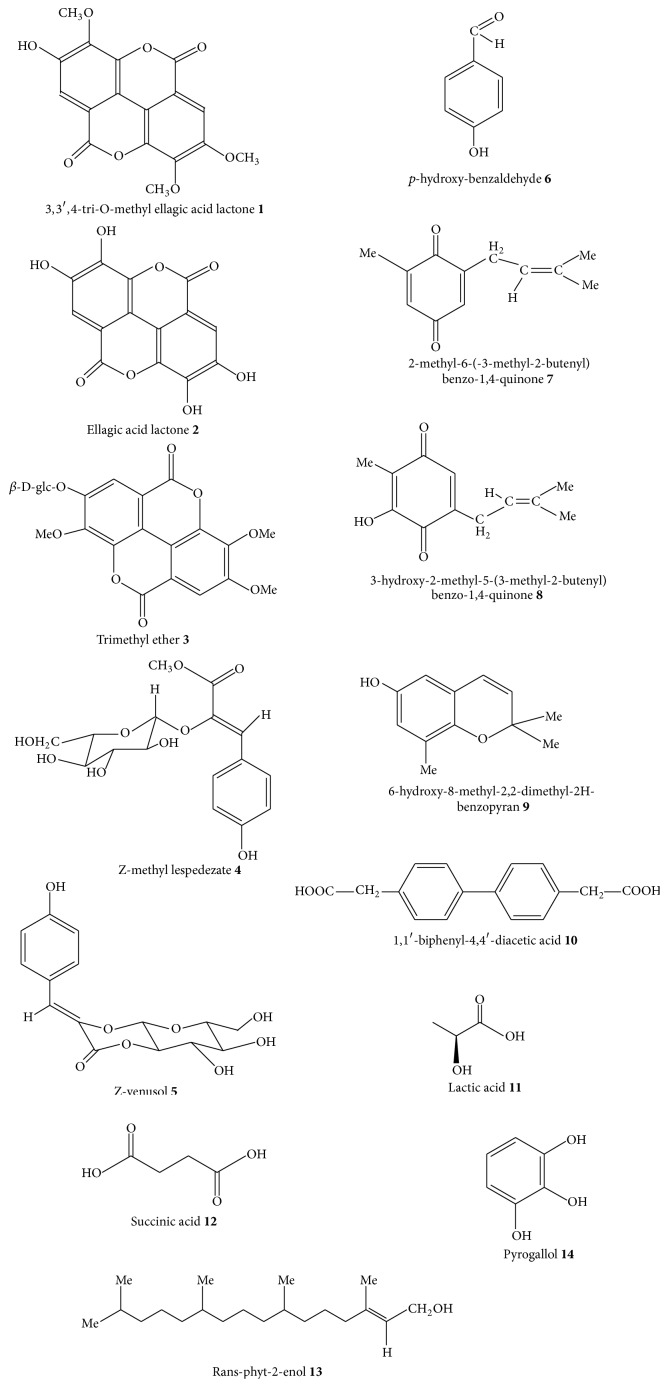
Chemical structures of compounds isolated from* Gunnera perpensa.*

Figure 1 shows structures of some of the secondary metabolites isolated from the leaves, rhizomes, roots, and stems of* G. perpensa*. These are the plant parts that are used to prepare* G. perpensa* herbal decoctions or infusions that are widely used in different traditional medicine systems in southern Africa. The reported compounds were identified and characterized by various criteria including UV, ^1^H NMR, ^13^C NMR, and mass spectroscopy. The phytochemical screening of methanolic extract of* G. perpensa* rhizomes carried out by Simelane et al. [54] revealed the presence of steroids, saponins, and glycosides in addition to secondary compounds shown in Figure 1. Mtunzi et al. [55] quantified inorganic elements in* G. perpensa* roots, with manganese showing the highest concentration of 1.46 ± 0.001 ppm, followed by iron (1.12 ± 0.003 ppm), nickel (0.239 ± 0.006 ppm), zinc (0.201 ± 0.0002 ppm), lead (0.153 ± 0.003 ppm), and copper (0.124 ± 0.002 ppm). According to Mtunzi et al. [55], the use of* G. perpensa *roots as herbal medicine will not cause heavy metal toxicity but can be of good use to the users in cases of micronutrient deficiency. Similarly, Chigor [56] isolated alkaloids, flavonoids, flavonols, phenols, proanthocyanidins, and tannins from aqueous, acetone, and methanol leaf and rhizome extracts of* G. perpensa*. Brookes and Dutton [57] isolated 3,3′,4′-tri-O-methyl ellagic acid lactone** 1**, ellagic acid lactone** 2**, Z-methyl lespedezate** 4**, p-hydroxy-benzaldehyde** 6**, 1,1′-biphenyl-4,4′-diacetic acid** 10**, and glucose from methanol extracts of* G. perpensa* roots. Drewes et al. [58] isolated 2-methyl-6-(-3-methyl-2-butenyl)benzo-1,4-quinone** 7**, 3-hydroxy-2-methyl-5-(3-methyl-2-butenyl)benzo-1,4-quinone** 8**, and 6-hydroxy-8-methyl-2,2-dimethyl-2H-benzopyran** 9** from dichloromethane extract of the leaves and stems of* G. perpensa* while rans-phyt-2-enol** 13** was isolated from methanol extracts of the aerial parts of the species. Khan et al. [59] isolated trimethyl ether of ellagic acid glucoside** 3**, Z-venusol, 7,8-dihydroxy-6-(hydroxymethyl)-3-[(Z)-(4-hydroxyphenyl)methylidene]tetrahydro-4aH-pyrano[2,3-b][1,4]dioxin-2-one** 5**, lactic acid** 11**, succinic acid** 12, **and pyrogallol** 14**, from the aqueous extract of the dry rhizomes of* G. perpensa*.

## 3. Pharmacological Activities

Some of the pharmacological activities of* G. perpensa *reported in literature correlate with some of its ethnomedicinal uses as a single agent or as part of a complex herbal decoction or infusion mixed with other plant species as summarized in Tables 1 and 2. While some of the listed pharmacological activities may not relate directly to the ethnomedicinal uses of* G. perpensa*, they may provide some insight into its potential therapeutic value and bioactive properties. The bioactive properties that have been reported so far based on* G. perpensa *crude extracts include acetylcholinesterase (AChE) enzyme inhibition [35, 60], anthelmintic [61, 62], antibacterial [27, 32, 58, 60, 63–65], antifungal [32, 58, 60, 63], antinociceptive [66], anti-inflammatory [58, 60, 65, 66], antioxidant [54, 65], antitumour [67], lactogenic [35], and uterotonic [21, 59] properties.

## 4. Acetylcholinesterase (AChE) Enzyme Inhibition

Ndhlala et al. [60] investigated the acetylcholinesterase enzyme inhibitory activity of aqueous, petroleum ether, dichloromethane, and 80% ethanol rhizome extracts of* G. perpensa* using the enzyme isolated from electric eels.* Gunnera perpensa* water extracts showed good AChE inhibitory activity (>90%) with IC_50_ value of 3.249 ± 0.56 *µ*g/mL which is considered potent inhibitor of AChE. Similarly, Simelane et al. [35] estimated the acetylcholinesterase activity of an aqueous extract of* Gunnera perpensa* rhizome using acetylthiocholine iodide and found the extract to inhibit 23% of AChE activity. Ozturk Sarikaya [68] evaluated the compound pyrogallol** 14** as a potential inhibitor for AChE enzyme and the results showed that the compound exhibited potent AChE enzyme inhibitory activity with IC_50_ and inhibitory constant (*K*_*i*_) values 10.2 and 8.6 *μ*M, respectively. These findings call for detailed research on acetylcholinesterase (AChE) enzyme inhibition activities of* G. perpensa* as the mechanisms of action of the species during muscle contraction when used as herbal medicine to induce labour or expel placenta after birth [15, 18, 22, 24–26, 35] are through the inhibition of AChE enzyme.

## 5. Anthelmintic

Victor and Keith [51] evaluated in vitro anthelmintic efficacy of* G. perpensa* aqueous leaf extract against* Heterakis gallinarum*. At a dosage of 29 mg/mL,* G. perpensa* had 60% worm motility inhibition at 12-hour interval and worm mortality index of 60% showing that the species has moderate anthelmintic properties. In another study, von Staden et al. [52] determined the anthelmintic efficacy of aqueous leaf extract of* G. perpensa* against* Heterakis gallinarum* in village chickens using the modified quantitative McMaster (floatation) technique [69] with distilled water as negative control and mebendazole as positive control. At days 7 and 14,* G. perpensa *had egg count reduction percentage ranging from 71 to 100%, signifying that the species has remarkable anthelmintic properties and has long-acting effects on* Heterakis gallinarum* in the system of the chickens. At 200 and 400 mg/kg doses,* G. perpensa *had worm count reduction of 78 and 74%, respectively [62].

Mwale et al. [70] evaluated the haematological and serum biochemical parameters of* G. perpensa* aqueous leaf extract in village chickens naturally infected with* Heterakis gallinarum*. From day 0 to 14, haematocrit was reduced for chickens orally administered with* G. perpensa *50, 100, and 400 mg/kg doses and haemoglobin was out of the range on day 0 and improved to be within the range on days 7 and 14. The observation that haemoglobin and haematocrit were within the expected range signifies that* G. perpensa* could influence the replenishment of lost blood thereby curbing anaemia that may be caused by* Heterakis gallinarum *[71]. According to Brookes and Dutton [57], 3,3′,4′-tri-O-methyl ellagic acid lactone** 1** which was isolated from* Combretum kraussii* Hochst. also demonstrated antihaemorrhagic properties. These documented anthelmintic properties correlate with the ethnomedicinal applications of* G. perpensa*, which is widely used as remedy for gastrointestinal parasites in Lesotho and South Africa [28, 31, 37].

## 6. Antibacterial

McGaw et al. [64] determined the antibacterial activity of* G. perpensa *roots and rhizomes against* Bacillus subtilis*,* Escherichia coli*,* Klebsiella pneumoniae, *and* Staphylococcus aureus* using the disc-diffusion assay and Neomycin (5 *µ*g) as positive control. Ethanol and water extracts of* G. perpensa *were active with MIC values of 3.13 and 0.78 mg/ml against* Staphylococcus aureus*, respectively. Steenkamp et al. [65] evaluated antibacterial activities of water and methanol extracts of dried roots of* G. perpensa* against* Escherichia coli, Pseudomonas aeruginosa, Staphyloccocus aureus, *and* Streptococcus pyogenes. *The methanol extract of* G. perpensa *presented MICs of 1 mg/ml, 2 mg/ml, and 4 mg/ml against* Staphylococcus aureus*,* Streptococcus pyogenes, *and* Escherichia coli*, respectively. The water extracts were less active with MIC values of 4 mg/ml and 2 mg/ml against* Streptococcus pyogenes *and* Staphylococcus aureus*, respectively [65]. Molares and Ladio [13] also evaluated antibacterial activity of* G. perpensa *rhizomes against the Gram-positive bacteria* Enterococcus faecalis *and* Staphylococcus aureus *and the Gram-negative bacteria* Escherichia coli *and* Pseudomonas aeruginosa*. A moderate to weak level of antibacterial activity in most of the extracts was reported, with the best minimal inhibitory concentration (MIC) value of 2.61 mg/ml shown by the acetone extract against* Staphylococcus aureus*. Based on these results, McGaw et al. [27] concluded that the relatively weak antibacterial activity is unlikely to justify the use of* G. perpensa *rhizomes in the traditional treatment of endometritis. Rather, the slightly antibacterial nature of the rhizomes may contribute to an additive effect, along with their known uterotonic activity, to the overall efficacy of the herbal decoction or infusion of the species [27]. Aqueous, ethanolic, and ethyl acetate extracts of* G. perpensa* roots were screened for antibacterial activity against* Bacillus subtilis, Escherichia coli*,* Klebsiella pneumoniae, *and* Staphylococcus aureus *by Buwa and Van Staden [32]. Results obtained by Buwa and Van Staden [32] revealed that the aqueous and ethanolic extracts of* G. perpensa *had the highest inhibitory activity against all the Gram-negative bacteria,* Escherichia coli *and* Klebsiella pneumoniae* with MIC values ranging from 0.78 to 1.56 mg/ml. Nkomo and Kambizi [63] also evaluated the antibacterial activity of methanol and water extracts of* G. perpensa *rhizomes against* Bacillus cereus, Escherichia coli, Klebsiella pneumoniae, Micrococcus kristinae, Pseudomonas aeruginosa, Serratia marcescens, Shigella flexneri, Staphylococcus aureus, Staphylococcus epidermidis, *and* Streptococcus faecalis. *The aqueous and methanolic extracts of* G. perpensa *were active against all the ten bacterial strains with MIC values ranging from 0.1 to 5 mg/ml. Similarly, Ndhlala et al. [60] evaluated the antibacterial activity of aqueous, petroleum ether, dichloromethane, and 80% ethanol rhizome extracts of* G. perpensa *against* Bacillus subtilis, Escherichia coli, Klebsiella pneumoniae,* and* Staphylococcus aureus *using the microdilution bioassay. The extracts of* G. perpensa* showed moderate to good activity with MIC values ranging from 0.195 to 12.5 mg/mL. Ethanol extracts showed best antibacterial activity ranging from 0.195 to 0.39 mg/mL while all water extracts had MIC values of 0.78 mg/mL. Muleya et al. [72] evaluated antibacterial activities of* G. perpensa* acetone, crude, dichloromethane, ethyl acetate, hexane, methanol, and water extracts against* Enterococcus faecalis*,* Escherichia coli*,* Pseudomonas aeruginosa, and Staphylococcus aureus* using the microdilution method with gentamicin and 70% acetone as positive and negative controls, respectively. Methanol fraction was the most active with an EC_50_ value of 80 *μ*g/ml against* Pseudomonas aeruginosa *[72]. In an earlier study, McGaw et al. [27] quantified antibacterial activity of crude extracts of stems, roots, and leaves of* G. perpensa* as well as 2-methyl-6-(-3-methyl-2-butenyl)benzo-1,4-quinone** 7**, 3-hydroxy-2-methyl-5-(3-methyl-2-butenyl)benzo-1,4-quinone** 8**, and 6-hydroxy-8-methyl-2,2-dimethyl-2H-benzopyran** 9** against* Bacillus cereus, Cryptococcus neoformans, Enterococcus faecalis, Escherichia coli, Klebsiella pneumoniae, Staphylococcus aureus,* and* Staphylococcus epidermidis* using ciproflaxin as control. McGaw et al. [27] obtained highest sensitivity from the leaf extracts followed by the stems, with the least activity noted for the root extracts. These findings corroborate reports that the leaves of* G. perpensa* which are used by the rural people of the Eastern Cape province, South Africa, in wound dressing [36] could be effective against bacterial infections. In the same study by McGaw et al. [27], 2-methyl-6-(-3-methyl-2-butenyl)benzo-1,4-quinone** 7** showed weak to moderate antibacterial activity with MIC value of 70 *µ*g/ml for* Cryptococcus neoformans*, 39 *µ*g/ml for* Enterococcus faecalis *and* Staphylococcus aureus and Bacillus cereus* (18 *µ*g/ml), and 9.8 *µ*g/ml for* Staphylococcus epidermidis*. Another compound, 3-hydroxy-2-methyl-5-(3-methyl-2-butenyl)benzo-1,4-quinone** 8**, showed no activity, while 6-hydroxy-8-methyl-2,2-dimethyl-2H-benzopyran** 9** showed very weak activity for most bacterial species with notable activity against* Bacillus cereus* and* Cryptococcus neoformans *with MIC values of 75 *µ*g/ml [27]. These findings support the use of* G. perpensa* against bacterial infections, for example, its traditional use against boils [43], endometrtitis [27], gonorrhoea [32], rheumatic fever [22, 24], syphilis [32], ulcers [73], and urinary tract infections [32].

## 7. Antifungal

Buwa and Van Staden [32] evaluated the antifungal activity of aqueous, ethanolic, and ethyl acetate root extracts of* G. perpensa* against* Candida albicans.* Results obtained by Buwa and Van Staden [32] revealed that the aqueous and ethyl acetate extracts of* G. perpensa *had weak to moderate inhibitory activity with MIC values of 25 and 6.25 mg/ml, respectively. Nkomo and Kambizi [63] evaluated the antifungal activity of methanol and water extracts of* G. perpensa *rhizomes against* Aspergillus flavas, Aspergillus niger, Candida albicans,* and* Penicillium notatum. *Results obtained by Nkomo and Kambizi [63] showed* G. perpensa *to be effective against* Penicillium notatum, Aspergillus flavas, *and* Aspergillus niger *with LC_50_ values ranging from 0.07 to 3.81. Similarly, Ndhlala et al. [60] investigated the antifungal activity of aqueous, petroleum ether, dichloromethane, and 80% ethanol rhizome extracts of* G. perpensa *against* Candida albicans *using the microdilution assay. The extracts of* G. perpensa *showed moderate to very good activity with MIC and MFC values ranging from 0.093 to 6.25 mg/mL with ethanol showing the best antifungal activity with MIC and MFC values of 0.093 mg/mL and 0.78 mg/mL, respectively. Muleya et al. [72] evaluated antifungal activities of* G. perpensa* acetone, crude, dichloromethane, ethyl acetate, hexane, methanol, and water extracts against* Candida albicans* and* Cryptococcus neoformans* using the microdilution method with gentamicin and 70% acetone as positive and negative controls, respectively. Methanol fraction was the most active with an EC* Candida albicans* and* Cryptococcus neoformans* of 160 *μ*g/ml against* Candida albicans *[72]. In an earlier study, McGaw et al. [27] quantified antifungal activity of crude extracts of stems, roots, and leaves of* G. perpensa* as well as 2-methyl-6-(-3-methyl-2-butenyl)benzo-1,4-quinone** 7**, 3-hydroxy-2-methyl-5-(3-methyl-2-butenyl)benzo-1,4-quinone** 8**, and 6-hydroxy-8-methyl-2,2-dimethyl-2H-benzopyran** 9** against* Candida albicans* using amphotericin B as control. McGaw et al. [27] obtained highest sensitivity from the leaf extracts followed by the stems, with the least activity noted for the root extracts. Compound, 3-hydroxy-2-methyl-5-(3-methyl-2-butenyl)benzo-1,4-quinone** 8**, showed no activity while 2-methyl-6-(-3-methyl-2-butenyl)benzo-1,4-quinone** 7** and 6-hydroxy-8-methyl-2,2-dimethyl-2H-benzopyran** 9** showed weak to moderate activity with MIC values of 130 and 37 *µ*g/ml, respectively [27]. These documented antifungal properties of* G. perpensa *justify its use as herbal medicine against microbial infections.

## 8. Antinociceptive and Anti-Inflammatory

Nkomo et al. [66] evaluated the antinociceptive and anti-inflammatory activities of aqueous and methanolic extracts of* G. perpensa* rhizome using the abdominal constriction, hotplate, formalin, hyperalgesia, and fresh egg albumin-induced inflammation. According to Nkomo et al. [66], both aqueous and methanolic extracts of* G. perpensa *demonstrated analgesic activities which were not dose dependent. In the acetic acid-induced writhing test, both doses of methanolic extracts of* G. perpensa* significantly reduced abdominal contortions. Nkomo et al. [66] used the hot-plate test to assess the central antinociceptive properties of* G. perpensa *with both doses of aqueous and methanolic extracts significantly increasing the reaction time to thermal stimulation. The formalin test induced a biphasic response in all animals, and during the inflammatory phase both the aqueous and methanolic extracts significantly reduced pain. These findings suggest that* G. perpensa *possesses both antinociceptive and anti-inflammatory activities supporting its traditional use for pain management. Ndhlala et al. [60] investigated the anti-inflammatory effects of aqueous rhizome extracts of Gunnera perpensa using Cyclooxygenase (COX-1 and COX-2) inhibitory bioassays. The water extracts of* G. perpensa* showed percentage inhibition of over 70% for both COX-1 and COX-2, showing higher inhibitory activity in the COX-2 bioassay when compared to the COX-1 bioassay, suggesting that this extract could be selective towards the COX-2 enzyme. The high COX-2 inhibitory activity of* G. perpensa* makes the species a better product when treating because the COX-2 enzyme is specific in treating inflamed tissue, resulting in less gastric irritation as compared to COX-1 inhibitors and hence decreased risk of gastric ulceration [60]. Similarly, Muleya et al. [72] evaluated anti-inflammatory activity of* G. perpensa* using the in vitro lipoxygenase inhibition assay determined by the soybean derived 15-lipoxygenase type I-B (15-LOX).* Gunnera perpensa *exhibited some soya bean 15-LOX inhibitory activity with EC_50_ value of 81.18 *μ*g/ml [72]. Lim et al. [74] evaluated anti-inflammatory and antinociceptive activities of* p*-hydroxy-benzaldehyde** 6** isolated from* Gastrodia elata* Blume using the acetic acid-induced vascular permeability test and acetic acid-induced writhing test in male ICR mice. The compound,* p*-hydroxy-benzaldehyde** 6**, suppressed the production of nitric oxide and induction of inducible nitric oxide synthase COX-2 in the lipopolysaccharide- (LPS-) activated RAW264.7 macrophages [74]. The compound,* p*-hydroxy-benzaldehyde** 6**, also diminished the reactive oxygen species level elevated in the LPS-activated macrophages [74].* Gunnera perpensa* can therefore be used for treating inflammation related conditions including abdominal pain, swelling of the body, menstrual pains, kidney inflammation and problems, sores, general body pain, and wounds (see Tables 1 and 2).

## 9. Antioxidant

Steenkamp et al. [65] evaluated the antioxidant activity of* G. perpensa *via oxidant generation by N-formyl-methionyl-leucylphenylalanine- (FMLP-) stimulated neutrophils measured using lucigenin-dependent chemiluminescence as described by Allen [75]. Extracts of* G. perpensa *showed possible scavenging activity in a concentration dependent manner with water extracts demonstrating higher activity than the methanol extracts as they significantly decreased luciginin enhanced chemiluminescence at concentrations of 100 *µ*g/ml and higher [65]. Simelane et al. [54] also evaluated the antioxidant activity of* G. perpensa* rhizome and found methanol extracts to exhibit strong scavenging of 2,2-diphenyl-1-picryl-hydrazyl (DPPH) and 3-ethylbenzothiazoline-6-sulfonate (ABTS) but showed poor (<50%) radical scavenging of nitric oxide, superoxide, and hydroxyl radicals. At a concentration of 5 mg/100 ml, the methanol extract was able to inhibit lipid peroxidation of the whole rat brain homogenate (71.13%) and lipoxygenase (30%) activity.* Gunnera perpensa* methanol extract also contained reduced form of nicotinamide adenine dinucleotide (NADH, 3.8 *ρ*m/g), total phenol (248.45 mg/g), and traces of sulfhydryl groups (SH). According to Simelane et al. [54] the total antioxidative capacity of* G. perpensa* was 36% relative to ascorbic acid (AA) and 64% relative to butylated hydroxyl toluene (BHT). Muleya et al. [72] assessed antioxidant scavenging capacity of* G. perpensa* acetone, crude, dichloromethane, ethyl acetate, hexane, methanol, and water extracts using 2,2-di (4-tert-octylphenyl)-1-picrylhydrazyl and 2,2′-azinobis (3-ethylbenzothiazoline)-6-sulfonic as substrate. The highest activity was obtained from methanol fraction of* G. perpensa *with EC_50_ value of 1.1 *μ*g/ml against 2,2-di (4-tert-octylphenyl)-1-picrylhydrazyl [72]. Similarly, Ozturk Sarikaya [68] studied different in vitro antioxidant assays such as cupric ion Cu^2+^ reducing power, Fe^3+^ reducing power, total antioxidant activity by ferric thiocyanate method, ABTS radical scavenging, DMPD radical scavenging, DPPH scavenging, Fe^2+^ chelating, O_2_^−^ scavenging, and H_2_O_2_ scavenging activities of the compound pyrogallol** 14**. The compound pyrogallol** 14** inhibited 78.0% lipid peroxidation of linoleic acid emulsion at 30 *μ*g/mL concentration; and BHA, BHT, *α*-tocopherol, and trolox exhibited inhibitions of at least 83.8% against peroxidation of linoleic acid emulsion at the same concentration [68]. In addition to this, pyrogallol** 14** was effective of all the scavenging and reducing power results [68]. Previous researchers argued that the antioxidant properties of* G. perpensa* are probably due to the presence of flavonoids and phenolics [56] as these compounds, for example, flavonoids, are known to have free radical scavenging capacity, coronary heart disease prevention, hepatoprotective, anti-inflammatory, antioxidative, anticancer, and antiviral activities [76].

## 10. Antitumour

Mathibe et al. [67] evaluated the in vitro antitumour effects of Z-venusol** 5** isolated from the roots of* G. perpensa *as well as Re-Joovena™, a commercial concoction containing* G. perpensa *(0.3 mg/ml),* Ocotea bullata *(Burch.) E. Meyer (0.3 mg/ml), and unspecified quantities of Vitamin E using human breast (MCF-7) cancer cells and human mammary epithelial cells (HMECs) with cisplatin and camptothecin drugs as positive controls. Z-venusol** 5** showed a statistically significant, concentration dependent, apoptotic inhibitory effect on proliferation of MCF-7 cells, with an IC_50_ of 53.7 *μ*g/ml after 72-hour exposure and the highest concentration (250 *μ*g/ml) used resulted in 69% inhibition [67]. There was insignificant inhibition (of 20%) of HMECs proliferation which was observed when concentration of Z-venusol** 5** was increased beyond 16.6 *μ*g/ml and the highest concentration used resulted in only 27% inhibition of proliferation of HMECs [67]. The fluorescein isothiocyanate Annexin V and the lactate dehydrogenase activity assays suggested that Z-venusol** 5** induced apoptotic cell death in the breast cancer cells. None of the Re-Joovena concentrations tested showed any significant effect. These findings suggest that Z-venusol** 5** is cytotoxic to human breast tumour cells in vitro, and cell death follows an apoptotic pathway. Khan et al. [77] evaluated the in vitro antiproliferative activity of pyrogallol** 14 **towards human tumour cell lines, including human erythromyeloid K562, B-lymphoid Raji, T-lymphoid Jurkat, and erythroleukemic HEL cell lines. In this study, inhibition of cell proliferation was consistently observed with IC_50_ values of pyrogallol** 14 **on K562, Jurkat, HEL, and Raji cell lines within the range of 10–30 *µ*M [77]. These documented antitumour properties of* G. perpensa *justify the use of gently warmed aqueous rhizome decoctions and infusions administered orally for three to four weeks as remedy for cancer in the Eastern Cape province, South Africa [78].

## 11. Lactogenic 

Simelane et al. [35] evaluated the effect of an aqueous extract of* G. perpensa* rhizome on milk production in rats. Female lactating rats that received oral doses of the extract of* G. perpensa *significantly produced more milk than controls. The mammary glands of rats treated with* G. perpensa* extract showed lobuloalveolar development and 0.8 *μ*g/ml of the extract was also found to stimulate the contraction of the uterus [35]. It is inferred that the plant extract exerted its activity on milk production and secretion by stimulating lobuloalveolar cell development and the contraction of myoepithelial cells in the alveoli. It is concluded that* G. perpensa *contains constituents with lactogenic activity mediated through binding to acetylcholine receptors that apparently contribute to its effectiveness in folk medicine. The reported lactogenic properties of* G. perpensa* corroborate the traditional use of the species to stimulate milk production in KwaZulu Natal province, South Africa [35].

## 12. Uterotonic

Kaido et al. [21] investigated the uterotonic activity of the crude decoction of* G. perpensa *on the isolated rat uterus and ileum preparation. Aqueous extract of* G. perpensa *initiated contractions in the isolated rat uterus, showed direct smooth muscle activity on the uterus, and potentiated the initial response of the uterus to oxytocin. Khan et al. [59] evaluated the effect of aqueous* G. perpensa *extract, ethyl acetate, ethyl acetate-methanol extract, and pure Z-venusol** 5** on rat uterine and ileal muscles.* Gunnera perpensa* extract stimulated direct contractile response on isolated uterine smooth muscle and induced a state of continuous contractility of the uterus once all physiological buffer had been removed from the organ bath. By contrast, Z-venusol** 5** did not trigger the direct contractile response but induced the state of continuous contractility once the organ bath was flushed [59]. These uterotonic properties of* G. perpensa* which promote uterine contractions were identified by traditional healers in southern Africa several years ago, and the species is now widely used to induce or augment labour, as an antenatal medication to tone the uterus and to assist in the expulsion of the placenta [15, 16, 18–22, 24–26, 35, 79].

## 13. Toxicity and Mutagenic

McGaw et al. [27] evaluated the possible toxicity of* G. perpensa* rhizome extracts using the brine shrimp microwell cytotoxicity bioassay [80]. All the extracts were lethal to the brine shrimp larvae at a concentration of 5 mg/ml. The acetone extract was extremely toxic at 1 mg/ml, with some toxicity evident at 0.1 mg/ml with the dichloromethane, ethanol, water, hexane, and methanol extracts displaying little activity at concentrations lower than 5 mg/ml [27]. Simelane et al. [54] evaluated the toxicity of* G. perpensa* rhizome methanol extract using the brine shrimp lethality test. The degree of the brine shrimp lethality was found to be directly proportional to the different concentrations of the extract, with lethal concentration (LC_50_) of 137.62 mg/100 ml [54]. Mwale and Masika [81] evaluated the potential toxicity of* G. perpensa *leaf aqueous extract through the acute, subacute, and chronic toxicity tests using Wistar rats. Neither rat mortality nor changes in behaviour were noted for acute test and rat mortality for 400 mg/kg dose of subacute and 200 mg/kg of chronic test was 20% [81]. The authors observed mild splenic siderosis and renal inflammation in the subacute test and therefore* G. perpensa* is potentially toxic when used consecutively for a long period. Simelane et al. [35] determined the cytotoxicity activity of aqueous rhizome extract of* G. perpensa *using the MTT cell proliferation assay via the human embryonic kidney (HEK293) and human hepatocellular carcinoma (HepG2) cells. The cytotoxicity of the extract (LC_50_) to two human cell lines (HEK293 and HepG2) was 279.43 *μ*g/ml and 222.33 *μ*g/ml, respectively [35].

The nontoxic nature of aqueous* G. perpensa *extracts has also been demonstrated at a cellular level using human fibroblast and monkey vero cell lines by Brookes and Smith [82]. Brookes and Smith [82] investigated whether* G. perpensa* exhibited any significant toxic effects on monkey vero cells and human fibroblasts. These cells were exposed for 24 hours to aqueous extracts of* G. perpensa* at concentrations ranging from 500 *μ*g/ml to 8 *μ*g/ml. The threshold for zero cell deaths occurred for monkey vero cells at 250 *μ*g/ml, and at this concentration it was found that 100% of human fibroblast cells survived [82]. The authors estimated the concentrations of* G. perpensa* in the bloodstream to be 4.6 *μ*g/ml, and based on this dilution that takes place in the bloodstream, the extract of this species is regarded as nontoxic.

Ndhlala et al. [60] investigated the mutagenic activity of aqueous rhizome extract of* G. perpensa* using the* Salmonella* microsome assay based on the plate-incorporation procedure with* Salmonella typhimurium* tester strain TA98, with and without metabolic activation. The results revealed that all the extracts were nonmutagenic towards the* Salmonella typhimurium* strain TA98 for the assay with and without S9 metabolic activation. The results obtained in this study offer supporting evidence for the safe use of these water extracts. However, animals or in vivo studies followed by human clinical trials are needed before* G. perpensa *herbal decoctions and infusions are recommended to induce labour, easy childbirth, and labour pains and expel the placenta.

## 14. Conclusion

Biological and pharmacological studies of various extracts and isolated compounds from* G. perpensa* confirmed acetylcholinesterase (AChE) enzyme inhibition, anthelmintic, antibacterial, antifungal, antinociceptive, anti-inflammatory, antioxidant, antitumour, lactogenic, and uterotonic activities. There is need to assess if* G. perpensa* has other chemical compounds such as anthocyanins, caffeic acid, ellagitannin, and quercetin that have been isolated from other* Gunnera* species and related genera [83]. A large number of the isolated compounds shown in Figure 1 have not been biologically tested; therefore, these compounds must be evaluated biologically in more detail. Further investigations should focus on the bioactive properties of these isolated compounds and their mechanisms of action aimed at illustrating the correlation between ethnomedicinal uses and pharmacological activities of various extracts of the species. Thus, more systematic research is required on* G. perpensa *compounds; their effects need to be further proved through additional animal experiments. Future research should combine the pharmacological effects, mechanisms of action, and clinical applications in assessing the efficacy of* G. perpensa *compounds and/or their extracts. Continued research on* G. perpensa *compounds, development, and discovery of pharmaceutical products and drugs from this species in the future will require more detailed studies in both the preclinical and clinical trials.

Future research should also focus on assessing toxicological aspects of the leaves, rhizomes, roots, and stems of* G. perpensa* as at present there is not enough systematic data about the pharmacokinetics and toxicity of this species, especially target-organ toxicity. More rigorous investigations should be done in the future investigating dosage range that is safe for humans and evaluations of target-organ toxicity. Such research work should be extended to focus on aspects of quality control to ensure safety and the fact that potentially toxic components of* G. perpensa *in herbal decoctions and infusions are kept below tolerance levels. Future research should also investigate any side effects that are associated with intake of* G. perpensa *herbal decoctions and infusions as a monotherapeutic agent or as an ingredient of complex herbal concoctions. Future research should also evaluate the combinational, additive, and synergetic effects associated with complex herbal concoctions that have* G. perpensa* as an ingredient.

The traditional usage of* G. perpensa *as herbal medicine to induce or augment labour, as an antenatal medication to tone the uterus and assist in the expulsion of the placenta and other ethnomedicinal uses as detailed in Tables 1 and 2, resulted in increased collection of its rhizomes and roots from the wild. The widespread usage of* G. perpensa *in southern Africa calls for conservation strategies and mechanisms for sustainable utilization of the species. McGaw et al. [27] obtained highest antibacterial activity from the leaf extracts followed by the stems, with the least activity noted for the root extracts. These findings provide a strong motivation for comparing the phytochemistry and biological activities of the leaves, stems, flowering stalks, flowers, and fruits with those of the rhizomes and the roots so as to justify the plant part substitution as a means of sustainable utilization of the species. Harvesting of* G. perpensa *rhizomes and roots means that the whole plant is removed resulting in reductions in the population size of the species.

## Figures and Tables

**Table 1:** Traditional herbal tonics of *Gunnera perpensa* mixed with other plant species.

Herbal preparation	Plant species composition and parts used	Ethnomedicinal uses	Country practised	References
Decoction	Roots of *Acorus calamus* L. taken with rhizome of *G. perpensa*	Impotence	South Africa	[24]

Decoction	Leaves of *Asclepias humilis* (E. Mey.) Schltr. and roots of *Scabiosa columbaria* L. taken with *G. perpensa*	Regulate menstrual cycle	Lesotho	[28, 43]

Decoction/infusion	Chopped *Crinum* spp. bulb mixed with a handful of pounded *G. perpensa *roots	Urinary complaints and pain in rheumatic fever	South Africa	[20]

Decoction/infusion	Roots of *Gladiolus sericeovillosus* Hook. f. subsp. *sericeovillosus* mixed with roots of *G. perpensa*	Administered to facilitate expulsion of the afterbirth	South Africa	[17]

*Inembe *decoction	Roots of *Cyphostemma natalitium* (Szyszyl.) J.J.M. van der Merwe, *Rhoicissus tridentata* subsp. *cuneifolia *(Eckl. & Zeyh.) Urton, and *Triumfetta rhomboidea* Jacq. mixed with roots of *G. perpensa*	Induce or augment labour, postnatal medication to expel afterbirth, abortifacient, administered to animals to expel placenta and treatment of endometritis	South Africa	[16, 20, 22–24, 27]

Infusion	Rootstock of *Alepidea amatymbica *Eckl. & Zeyh. var. *amatymbica *is mixed with that of *G. perpensa*	Stomachache	South Africa	[20]

Infusion	Bark of *Cassine transvaalensis *(Burtt Davy) Codd taken with rhizome of *G. perpensa*	Psoriasis	South Africa	[84]

*Imbiza ephuzwato decoction*	*Acokanthera oppositifolia* (Lam.) Codd (roots), *Aster bakeranus* Burtt Davy ex C.A. Sim. (roots), *Corchorus asplenifolius *Burch. (roots), *Cyrtanthus obliquus* (L.f.) Aiton (bulb), *Drimia elata* Jacq. (bulbs), *Eriosema cordatum* E. Mey. (roots), *Fusifilum physodes* (Jacq.) Raf. ex Speta (bulbs), *Gnidia kraussiana* Meisn. var. *kraussiana* (roots), *Gomphocarpus fruticosus* (L.) W.T. Aiton (roots), *G. perpensa* (rhizomes), *Hypericum aethiopicum* Thunb. (leaves, stems), *Ledebouria *spp. (bulbs), *Lycopodium clavatum* L. (whole plant), *Momordica balsamina* L. (leaves), *Rubia cordifolia* L. (roots), *Scadoxus puniceus* (L.) Friis & Nordal (bulb), *Stephania abyssinica* (Quart.-Dill. & A. Rich.) Walp. (roots), *Tetradenia riparia* (Hochst.) Codd (leaves), Vitellariopsis marginata (N.E. Br.) Aubrév (roots), Watsonia densiflora Bak. (corms), and *Zanthoxylum capense* (Thunb.) Harv. (roots)	A detoxifying and energizing tonic used to increase sexual prowess and relieve constipation, stress, high blood pressure, arthritis, kidney problems, and general body pains	South Africa	[60]

*Isihlambezo* decoction	*Agapanthus africanus *(L.) Hoffmans (roots), *Callilepis laureola *DC. (roots), *Clivia miniata* (Lindl.) Bosse (leaves), *Combretum erythrophyllum *(Burch.) Sond. (roots), *Crinum* spp. (bulb), *Gomphocarpus fruticosus *(L.) W.T. Aiton (roots), *G. perpensa* (rhizomes), *Gymnanthemum corymbosum* (Thunb.) H. Rob. (roots), *Pentanisia prunelloides* (Klotzsch) Walp. (roots), *Rhoicissus tridentata *subsp. *cuneifolia* (roots), *Scadoxus puniceus* (bulb), and *Typha capensis *(Rohrb.) N.E.Br. (rhizome)	Used to induce or augment labour and as postnatal medication to expel the afterbirth, administered to animals to expel the placenta and treatment of endometritis	South Africa	[16, 20, 22–24, 27]

Re-Joovena	A concoction containing *G. perpensa* (0.3 mg/ml), *Ocotea bullata* (Burch.) E. Meyer (0.3 mg/ml), and unspecified quantities of Vitamin E	Used to treat haemorrhoids, pregnancy-related complications, painful breasts during menstruation, and management of several inflammatory disorders	South Africa	[67]

**Table 2:** *Gunnera perpensa* used as a single agent for various human and animal diseases and ailments in southern Africa.

Ethnomedicinal use	Plant parts used and administration	Country practised	References
Abdominal pain	Root decoction taken as tonic	South Africa	[24]

Bladder problems	Root decoction taken orally	South Africa	[29]

Bleeding stomach	Root decoction taken as tonic	South Africa	[22, 24]

Body cleansing	Leaves, root decoction taken orally	Lesotho; South Africa	[29, 53]

Boils	Leaves used as poultices	Lesotho	[43]

Cancer	Leaves, rhizome decoction or infusion taken orally	Lesotho; South Africa	[53, 78]

Colds	Root decoction taken orally	South Africa	[24, 79]

Constipation	Stem decoction taken orally	South Africa	[39]

Dysmenorrhoea	Root decoction taken orally	Lesotho; South Africa; Swaziland	[22, 24–26, 42–44, 63, 85]

Earache	Rhizome decoction applied topically	South Africa	[30]

Endometritis	Rhizome decoction taken orally	South Africa	[27]

Expel placenta after birth in humans and animals	Rhizome decoction taken orally	Lesotho; South Africa	[15, 18, 22, 24–26, 35]

Food	Leaves edible as leafy vegetables. Roots, stalks, and stems edible and used for making beer	South Africa; Swaziland	[40–42]

Gastrointestinal parasites	Rhizome decoction taken orally	Lesotho; South Africa	[28, 31, 37]

Gonorrhoea	Root decoction taken orally	South Africa	[32]

Headache	Leaves burnt and crushed and smoked, root decoction taken orally	Lesotho; South Africa	[43, 86]

Heart diseases	Root decoction taken orally	Lesotho	[33]

Hypertension	Root decoction taken orally	Lesotho	[33]

Impotence	Root decoction taken orally	South Africa; Swaziland	[16, 24, 42, 85]

Indigestion	Root decoction taken as tonic	South Africa	[24, 73]

Induce or augment labour and as antenatal medication	Rhizome decoction taken orally	South Africa	[22]

Infertility in women	Root decoction taken orally	South Africa	[29]

Kidney problems	Root decoction taken orally	South Africa	[29]

Poor appetite	Root decoction taken as tonic	South Africa	[24]

Psoriasis	Root infusions applied topically	South Africa	[22]

Pulmonary ailments	Rhizome decoction taken orally	South Africa	[73]

Rheumatic fever	Root decoction taken as tonic	South Africa	[22, 24]

Scabies	Root decoction taken as tonic	South Africa	[24]

Stimulate milk production	Root decoction taken orally	South Africa	[35]

Stomachache	Leaves, rhizome decoction taken orally	Lesotho; South Africa	[22, 53, 86]

Syphilis	Root decoction taken orally	South Africa	[32]

Swelling	Rhizome decoction applied topically	South Africa	[22]

Ticks and other parasites	Leaves and rhizome used as repellent	South Africa	[79]

Ulcers	Leaf decoction taken orally	South Africa	[73]

Urinary infections	Root decoction taken orally	South Africa	[32]

Urinary stones	Root tinctures	South Africa	[22]

Uterine bleeding	Root bark decoction taken orally	Swaziland	[87, 88]

Wound dressing	Leaves used as poultices, rhizome decoction applied as a dressing	Lesotho; South Africa	[16, 22, 24, 36, 43, 73]
